# Intestinal permeability in children/adolescents with functional dyspepsia

**DOI:** 10.1186/1756-0500-7-275

**Published:** 2014-05-01

**Authors:** Nancy A Neilan, Uttam C Garg, Jennifer Verrill Schurman, Craig A Friesen

**Affiliations:** 1 Division of Pathology and Laboratory Medicine, Children’s Mercy Hospitals & Clinics, Kansas City, USA; 2 Division of Developmental and Behavioral Sciences, Children’s Mercy Hospitals & Clinics, Kansas City, USA; 3 Division of Gastroenterology, Hepatology, & Nutrition, Children’s Mercy Hospitals & Clinics, 2401 Gillham Road, Kansas City, MO 64108, USA

**Keywords:** Functional dyspepsia, Intestinal permeability, Eosinophilic duodenitis, Sugar absorption test

## Abstract

**Background:**

An altered intestinal mucosal barrier has been demonstrated in subsets of patients with IBS and FAP and may be an additional biological factor contributing to symptom generation in children with FD. The objective of this study was to determine if intestinal permeability is increased in children/adolescents with functional dyspepsia (FD) and whether intestinal permeability is correlated with mucosal inflammation and/or symptoms of anxiety or depression in this population.

**Methods:**

A sugar absorption test was performed in 19 patients with FD and 19 controls. Anxiety and depression were assessed in both groups utilizing a standard questionnaire. In FD patients, duodenal mean and peak mast cell and eosinophil densities were determined.

**Results:**

Intestinal permeability as measured by the sugar absorption test did not differ between children with FD and controls. In children with FD, there was no correlation between permeability and mast cell density, eosinophil density, anxiety scores, or depression scores, respectively.

**Conclusions:**

Pediatric FD does not appear to be associated with increased small bowel intestinal permeability, however, there are some limitations to the current study.

**Trial registration:**

ClinicalTrials.gov; NCT00363597.

## Background

Recurrent abdominal pain is a common complaint among school-age children, being present in 13 to 17% at any given time [1]. It represents the most common chronic pain entity in pediatric patients. The great majority of these patients can be diagnosed with a functional gastrointestinal disorder (FGID) [2,3]. As established by Rome III, there are four FGIDs related to abdominal pain in children including irritable bowel syndrome (IBS), functional dyspepsia (FD), abdominal migraines, and functional abdominal pain (FAP) [4]. FD is defined as upper abdominal pain or discomfort unrelieved by defecation and in the absence of a structural or biochemical explanation for the pain [4]. FD, alone or in combination with irritable bowel syndrome, is present in 45-87% of children/adolescents referred to pediatric gastroenterologists for evaluation of chronic abdominal pain [1-3].

An altered intestinal mucosal barrier has been demonstrated in subsets of patients with IBS and FAP and may be an additional biological factor contributing to symptom generation in children with FD [5-7]. Barrier dysfunction results from alteration of the tight junctions between epithelial cells which normally control the passage through the paracellular space. Barrier dysfunction allows greater antigenic exposure and can facilitate mucosal inflammation. To our knowledge, intestinal permeability associated with barrier dysfunction has not been previously evaluated in patients with FD. However, barrier dysfunction can result from several factors which may be relevant to FGIDs, including FD, such as chronic stress, bacterial antigens, intestinal anaphylaxis, mast cell activation and cytokines related to allergic or T helper 2 (TH2) responses [5]. Intestinal barrier dysfunction has been demonstrated in children with food allergy even when asymptomatic on a food elimination diet [8].

This study was an initial exploration of the presence of intestinal permeability in a pediatric population diagnosed with FD. The specific aims of this study were as follows: 1) to compare small bowel permeability between children with FD and healthy children; 2) to explore the relationship between intestinal permeability and mucosal eosinophil and mast cell densities, respectively, in children with FD; and, 3) to explore relationships between intestinal permeability and anxiety and depression scores, respectively, for children with FD and healthy children. Understanding these relationships would help clarify the role of intestinal permeability in symptom generation in FD, as well as provide information on potential additional targets for treatment in this population.

## Methods

### Participants

Patients were candidates for inclusion in this study if they had been diagnosed with functional dyspepsia who were aged 8–17 years (inclusive), had a demonstrated a lack of clinic response to acid-reduction therapy, and were undergoing endoscopy to evaluate FD. Patients were excluded from the study if any of the following criteria were present: 1) previous abdominal surgery; 2) any chronic non-gastrointestinal illness requiring regular medical care (e.g., diabetes mellitus, juvenile rheumatoid arthritis, cystic fibrosis, cancer); 3) any history of an adverse reaction to lactulose or mannitol; 4) any use of aspirin within one week prior to the study; 5) any use of antacids or laxatives within 1 week prior to the study; 6) any use of steroids, antihistamines or antihistamine-like drugs within 4 weeks prior to the study; 7) any use of antibiotics within 4 weeks prior to the study; 8) pregnancy; or, 9) non-English speaking. Fifty-five patients were approached and 22 patients agreed to participate in the study, yielding a 40% recruitment rate. Three patients who participated in the study demonstrated pathology on biopsy and were excluded from data analysis, leaving 19 participants in the patient group (13 females, 6 males; mean age = 13 ± 2.5 years). Of these, one patient also was found to have eosinophilic esophagitis on biopsy, but was retained in the study as this was not an exclusion criterion.

For the control group, all respondents aged 8–17 years (inclusive) were candidates for inclusion in the study. In addition to the exclusion criteria noted above, control participants were not admitted to the study if there was any history of gastrointestinal symptoms (including abdominal pain or discomfort, nausea, vomiting, bloating, diarrhea, or constipation) within the previous six months. Twenty-four potential control participants responded to the study advertisement. Twenty-one of the respondents met eligibility criteria. Of these, two refused, leaving 19 controls enrolled in the study (10 females, 9 males; mean age = 13 ± 2.6).

### Measures

#### Intestinal permeability

Intestinal permeability is measured by evaluating absorption of macromolecules which are not typically absorbed across intestinal cells, but may pass through the paracellular space when tight junction disruption is present [5]. Lactulose is a non-digestible sugar and is absorbed through the paracellular space following damage to the tight junction; it is then rapidly metabolized by colonic bacteria upon entry into the colon.As such, lactulose often is utilized to test intestinal permeability as part of a Sugar Absorption Test (SAT) [5]. Mannitol is a monosaccharide that is readily absorbed through the transcellular pathway and is used to control for mucosal (e.g. intestinal surface area) and non-mucosal (e.g. gastric empting, intestinal transit, and impaired renal function) factors which may affect lactulose absorption and metabolism. Sucrose is added to make a hyperosmolar solution which has been shown to also improve sensitivity of the test. In the SAT procedure, urine is collected for 5 hours following administration of an oral lactulose/mannitol/sucrose solution. The concentration of lactulose and mannitol in the urine is determined and results are expressed as the ratio of lactulose to mannitol. An increased ratio indicates increased paracellular permeability due to tight junction dysfunction.

Urinary excretion of lactulose and mannitol was determined in the Toxicology Laboratory, CMH, KC, MO 64108 using an enzymatic method (available from INstruchemie, AC Delfzijl, The Netherlands) adapted to the V-Twin automated chemistry analyzer. Recovery of the sugar probes were expressed both quantitatively and as the lactulose/mannitol ratio. A urine creatinine was determined in order to express quantitative results in mmol/mol creatinine. Urine creatinine was performed in the Toxicology Laboratory, Children’s Mercy Hospital using the V-Twin automated chemistry analyzer.

#### Gastrointestinal eosinophils and mast cells

Eosinophil density was determined from the routine histology slides (hematoxylin and eosin stain) by counting eosinophils beginning in what appeared to be the most involved area after scanning the entire specimen. Five consecutive fields (400× magnification) were evaluated. Peak count was defined as the highest count of the five fields, while mean count was defined as the average of the five fields.

Mast cell density was evaluated utilizing immunohistochemical techniques. Serial 3-μm paraffin sections were air dried and heat fixed on slides. The sections were deparaffinized with xylene and iodine and rehydrated in a graded series of alcohol. Sections were stained on an automated Dako Autostainer 3400 using Dako’s LSAB + kit with streptoavidin conjugated to horseradish peroxidase. The antibody utilized was tryptase monoclonal mouse antihuman mast cell tryptase, clone AA1, Dako. Mast cell density was determined by counting mast cells beginning in what appeared to be the most involved area after scanning the entire specimen. Five consecutive fields (400× magnification) were evaluated. Peak count was defined as the highest count of the five fields, while mean count defined as the average of the five fields.

#### Anxiety and depression

Symptoms of anxiety and depression were assessed using the Behavioral Assessment System for Children (BASC) [9]. The BASC is a paper-and-pencil questionnaire assessing psychosocial functioning in youth with different versions available for children (ages 8–11 years), adolescents (ages 12–18 years), and parents (different versions for parents of children 6–11 years and 12–18 years). The BASC has demonstrated criterion-related and construct validity, has good internal consistency for most individual subscales, and is widely used in both clinical and research settings [9]. Standardized T scores for anxiety and depression were used for the present study.

#### Procedures

This pilot study was a single-site, case-controlled, single-blind observational design. Participants for the patient group were recruited in a single hospital-based clinic specializing in the evaluation and treatment of children with chronic or recurrent abdominal pain. Participants for the control group were recruited by advertisement at the same hospital. Informed parental permission and participant assent were obtained prior to the initiation of any study procedures.

The study involved one visit to the hospital-based gastroenterology clinic. After providing informed parental permission and participant assent, all participants completed the Behavior Assessment System for Children (BASC) and then underwent a differential sugar absorption test (SAT) using lactulose/mannitol/sucrose solution to evaluate intestinal permeability. Participants emptied their bladders immediately prior to the start of the SAT and then ingested 2 mL/kg (maximum 100 ml) of the sugar solution. The SAT solution contained 5 grams lactulose, 2 grams mannitol and 40 grams sucrose dissolved in demineralized water for a total volume of 100 mL. Participants did not eat or drink for 2 hours following ingestion of the sugar solution. After 2 hours, participants could consume any fructose-free foods and were asked to consume at least 1 cup of water hourly. Urine was collected for 5 hours following administration of the lactulose/mannitol solution. Finally, for participants in the patient group only, an endoscopy with biopsy was performed on a separate day as part of standard care and a minimum of 4 mucosal biopsies were obtained from the duodenum for later evaluation.

All study personnel performing procedures and assessing outcomes were blinded to group assignment. The study protocol was approved by the Children’s Mercy Kansas City Institutional Review Board.

#### Sample size

A priori analysis provided a sample size estimate of 18 per group which would allow 80% power to detect a difference for mean permeability between 0.015 and 0.045 assuming a standard deviation of 0.03.

#### Statistical analysis

Independent-sample t- tests (2-tailed) were used to evaluate mean differences in the lactulose/mannitol ratio in the patient group compared to the control group. In the FD patients, Pearson correlation coefficients were calculated and used to evaluate associations between the lactulose/mannitol ratio and mast cell and eosinophil densities, as well as the BASC anxiety and depression subscale scores, respectively.

SPSS 18.0 for Windows computer program, SPSS Inc. Chicago, IL was used to complete the analyses. All analyses used a set significance limit of α = 0.05.

## Results

### Intestinal permeability in FD versus controls

Mean values for the lactulose/mannitol ratio (L/M) did not differ between FD patients and controls (0.034 ± .01 vs. 0.032 ± .01, p = .141). Only one FD patient had a L/M ratio exceeding the mean plus two standard deviations for the control subjects.

### Intestinal permeability in relation to mast cell and eosinophil densities

Mean mast cell density ranged from 7.2 to 27.6 cells/hpf (mean 16.0) and peak mast cell density ranged from 8 to 34 cells/hpf (mean 22.0). Mean eosinophils ranged from 7.2 to 54.2 cells/hpf (mean 24.6) and peak eosinophils ranged from 12 to 76/hpf (mean 38.8). There were no significant correlations between L/M and mast cell or eosinophil densities.

### Intestinal permeability in relation to anxiety and depression

Mean values for anxiety differed significantly between FD patients and controls for parent-report (55.8 ± 11.0 vs. 46.4 ± 10.0, p = .01), but not for self-report (51.2 ± 11.2 vs. 44.7 ± 9.2, p = .06). Mean values for depression also differed significantly between FD patients and controls for parent-report (51.4 ± 9.5 vs. 44.7 ± 9.0, p = .03), but not for self-report (47.1 ± 5.7 vs. 44.6 ± 2.5, p = .09). There were no significant correlations between L/M and scores on the anxiety or depression subscales based on parent- or self-report.

## Discussion

In the current study, FD does not appear to be associated with abnormal small bowel permeability. This stands in contrast to previous findings supporting alteration in the intestinal mucosal barrier in IBS and FAP. This suggests that either FD is not associated with altered permeability or perhaps, if there is a permeability abnormality, the anatomic sight is different from these other FGIDs. This latter explanation remains a possibility given that anatomic differences have been noted between FD and IBS with regards to location for inflammation, dysmotility, and visceral sensitivity.

The intestinal barrier is highly regulated by immune factors and increased permeability has been seen in other conditions associated with inflammation, such as Crohn’s disease and Celiac disease [10-13]. Mucosal eosinophilia has been implicated as a potentially significant factor in FD. Talley and colleagues found that FD in adults is associated with a significant increase in duodenal eosinophils as compared to controls [14]. Consistent with this, our own research group found duodenal mucosal eosinophilia (defined as a peak count of ≥20 eos/hpf) in 79% of children undergoing endoscopy with mucosal biopsy for evaluation of FD [15]. FD also has been associated with a significant increase in antral mast cells in adults [16]. Importantly, barrier dysfunction is associated with increased IL-4 and decreased IFN-δ expression in the gut mucosa (consistent with a TH2 response) [17]. Thus, the potential relationship between intestinal permeability and inflammation, as defined by mast cell and eosinophil densities, also was explored. However, no relationship was found, seeming to indicate that altered permeability is not a mechanism by which mast cells or eosinophils contribute to symptom generation in FD. However, it also is possible that the inflammation in FD is limited to a short enough segment of the proximal gastrointestinal tract that the L/M ratio, influenced by permeability across the entire small bowel, may not be a sensitive enough measure. There may be value in future assessment of the relationship between structural proteins related to permeability, such as zonulin or occludin, and inflammatory cell density in the proximal small bowel of FD patients.

Finally, there is an increasing body of evidence that stress, both acute and chronic, affects intestinal barrier function [18-20]. This pathway appears to be mediated by the release of corticotropin releasing hormone with subsequent activation of mucosal mast cells [19,21]. In the current study, however, no correlation was found between intestinal permeability and scores for anxiety or depression, respectively. Alteration of the mucosal barrier as indicated by the L/M ratio does not appear to be the mechanism by which anxiety and depression contribute to symptom generation in FD.

### Limitations

The sample size chosen for the current study was calculated to be able to detect a 3-fold difference in the L/M ratio. It is possible that a lesser difference would be physiologically relevant and that the sample size was too small to detect such differences. Although we evaluated for allergic inflammation (i.e. eosinophil and mast cell density) in the FD patients, a deficiency in the current study was that history regarding food allergy was not obtained in either FD patients or controls. It is possible that the effects of food allergy on permeability are independent of eosinophil and mast cell density. Lastly, it is possible that permeability abnormalities, if present in FD, are more localized to the duodenum and therefore not reflected by the sugar absorption test.

## Conclusions

Increased small bowel permeability does not appear to be a biomarker of FD in children even in the presence of elevated mast cell density, eosinophil density, anxiety scores or depression scores. However, given the limitations of the current study, future studies will be needed with a larger population, controlling for food allergy, and assessing whether more focal barrier disruptions are present (e.g. utilizing a Ussing chamber) before making any definitive conclusion regarding a potential role for barrier dysfunction in the pathophysiology of FD.

## Abbreviations

FGID: Functional gastrointestinal disorder; IBS: Irritable bowel syndrome; FD: Functional dyspepsia; FAP: Functional abdominal pain; SAT: Sugar absorption test; BASC: Behavioral assessment system for children; ROC: Receiver operating characteristic; L/M: Lactulose/mannitol ratio.

## Competing interests

The authors declare that they have no competing interests.

## Authors’ contributions

All authors contributed to study design, data analysis, and manuscript writing. NN and UG developed and/or performed laboratory methods. All authors read and approved the final manuscript.
